# Disheveled Hair and Ear (*Dhe*), a Spontaneous Mouse *Lmna* Mutation Modeling Human Laminopathies

**DOI:** 10.1371/journal.pone.0009959

**Published:** 2010-04-01

**Authors:** Paul R. Odgren, Craig H. Pratt, Carole A. MacKay, April Mason-Savas, Michelle Curtain, Lindsay Shopland, Tsutomu Ichicki, John P. Sundberg, Leah Rae Donahue

**Affiliations:** 1 Department of Cell Biology, University of Massachusetts Medical School, Worcester, Massachusetts, United States of America; 2 Institute for Molecular Biophysics, Bar Harbor, Maine, United States of America; 3 Genetic Resource Science, Bar Harbor, Maine, United States of America; 4 The Jackson Laboratory, Bar Harbor, Maine, United States of America; Emory University, United States of America

## Abstract

**Background:**

Investigations of naturally-occurring mutations in animal models provide important insights and valuable disease models. Lamins A and C, along with lamin B, are type V intermediate filament proteins which constitute the proteinaceous boundary of the nucleus. *LMNA* mutations in humans cause a wide range of phenotypes, collectively termed laminopathies. To identify the mutation and investigate the phenotype of a spontaneous, semi-dominant mutation that we have named Disheveled hair and ear (*Dhe*), which causes a sparse coat and small external ears in heterozygotes and lethality in homozygotes by postnatal day 10.

**Findings:**

Genetic mapping identified a point mutation in the *Lmna* gene, causing a single amino acid change, L52R, in the coiled coil rod domain of lamin A and C proteins. Cranial sutures in *Dhe*/+ mice failed to close. Gene expression for collagen types I and III in sutures was deficient. Skulls were small and disproportionate. Skeletons of *Dhe/*+ mice were hypomineralized and total body fat was deficient in males. In homozygotes, skin and oral mucosae were dysplastic and ulcerated. Nuclear morphometry of cultured cells revealed gene dose-dependent blebbing and wrinkling.

**Conclusion:**

*Dhe* mice should provide a useful new model for investigations of the pathogenesis of laminopathies.

## Introduction

The nuclear lamina (NL) is the proteinaceous boundary of the nucleus in metazoa. It separates the inner nuclear membrane from the chromatin. It is comprised of a meshwork of 10 nm filaments of a special group of class V intermediate filament proteins called the lamins, forming a “basket” surrounding the chromatin. It also includes a growing list of lamin-associated proteins [1]. There are lesser amounts of lamins in the nuclear interior. The NL also accommodates the nuclear pores, large complexes that span both the inner and outer nuclear membranes and control the import and export of macromolecules.

Lamins are divided into two broad classes, the B-type lamins and the A-type lamins, named for the genes from which they are transcribed. In mammals, the lamins are transcribed from 3 genes encoding lamins B1 and B2 (each transcribed from its own gene), and lamins A and C, both transcribed from the *LMNA* gene, but differentially spliced at the 3′ end to produce the two proteins [2], [3], [4]. Most adult mammalian cells contain all four of these lamins as well as 3 minor lamins, AΔ10, C2 (from *LMNA* alternative splicing), and B3 (via *LMNB2* alternative splicing). B-type lamins are essential for life, and during mitosis they remain associated with membrane vesicles that will reconstitute the nuclear envelope in daughter cells, whereas the A-type lamins disperse in the cytoplasm and re-enter the nucleus after it re-forms. Expression of the various lamins is developmentally regulated, with only B1 being present in early embryonic cells. It is thought that A-type lamins assist in differentiated cell functions. A-type lamins are absent from developing mouse nuclei before implantation and first appear about day 9 in visceral endoderm and trophoblast cells [5]. All lamins self-associate via parallel α-helical coiled-coil interactions of their central rod domains. They have short (20–30 amino acid) N-terminal non-helical domains, then an extended (nearly 400 amino acid) coiled coil domain, and a globular C-terminal region (roughly 250 amino acids).

Interest in the *LMNA* gene and in NL-associated proteins has grown dramatically since a number of mutations in the *LMNA* gene were shown to cause a wide range of connective tissue, neuropathological, and premature aging (progeria) syndromes in human patients (reviewed in [6]). The term “laminopathy” was coined to describe these genetic disorders, and that term was expanded to “nuclear envelopathies” to encompass similar syndromes caused by mutations in other proteins of the nuclear envelope [7]. An exemplar of laminopathy/envelopathy is Emery-Dreifuss muscular dystrophy (EDMD), which is autosomal dominant when caused by mutations in *LMNA*, whereas it is X-linked when caused by changes in the NL-associated protein, emerin.

The phenotypes associated with mutations in human *LMNA* encompass defects in adipocytes, cardiac and skeletal muscle, peripheral nerves, skin, and other tissues. Skeletal involvement is reported in progeria, in which bones are hypomineralized, clavicles are dysplastic, and joints are defective [8]. It is also a prominent feature of autosomal recessive mandibuloacral dysplasia (MAD), in which patients exhibit hypoplasia of mandibles and clavicles, delayed closure of sutures, facial abnormalities, and loss of distal bone from the extremities, along with lipodystrophy, mottled skin, and post-natal growth retardation [9]. A recent case report described a 56-year-old patient with MAD complicated by generalized osteolysis, leading to fractures, including of the vertebrae, resulting in paraplegia [10].

We report here a new semi-dominant spontaneous mouse mutation in the BXD8/TyJ strain with craniofacial defects, superficial abnormalities of the pinnae, and an abnormal, rough hair coat with epidermal dysplasia, called Disheveled hair and ear (*Dhe*). Closer examination revealed cranial dysostosis and reduced bone mineral density. We describe nuclear abnormalities, and the phenotype of mice heterozygous and homozygous for the *Dhe* mutation, plus identification of the underlying mutation in the *Lmna* gene, thus making *Dhe* (B6.BXD8-*Lmna^Dhe^*/TyJ) the first spontaneous *Lmna* mutation identified in the mouse.

## Results

### Initial phenotypic observations

**Figure 1:**
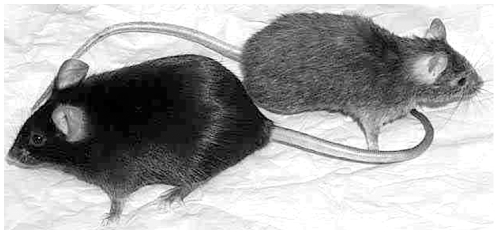
+/+ (left) and *Dhe/+* (right) mice at 12 weeks of age. Note the relatively sparse and gray coat, smaller overall size, and smaller pinnae of the mutant.

Heterozygous *Dhe/+* mice are smaller than normal littermates, have a sparse, scruffy coat, and short ear pinnae (**Figure 1**). Less apparent features include an underdeveloped lower jaw (inferior brachygnathism), protruding eyes due to shallow orbits (exopthalmous), malocclusion, low bone mineral density, reduced body fat, and flaky skin. The trunkal hair of heterozygous mice prematurely turns gray around 12 weeks of age; however, lifespan is comparable to wild type controls. Heterozygous males are reliable breeders, females less so. Homozygous *Dhe/Dhe* pups die at about 10 days of age and exhibit a more severe cranial phenotype than heterozygous littermates.

### Mapping the mutation and identifying a destabilizing Lmna mutation

Initial mapping of the *Dhe* mutation was performed in Dr. Benjamin Taylor's laboratory at The Jackson Laboratory (JAX) in 1994, using the MEV multiple ecotropic provirus linkage testing stock [11], [12], and was found to be on mouse Chromosome 3 (Chr 3) near D3Mit12. Subsequently, using backcross 1 mice from a cross between *Dhe/+* and CAST/EiJ, the candidate region was reduced to a 3.7 cM interval between MIT markers D3Mit74 and D3Mit49, position 85.9 and 89.6 Mb, respectively (National Center for Biotechnology Information (NCBI) build 36). No recombinants were found in 256 mutant mice.

**Figure 2:**
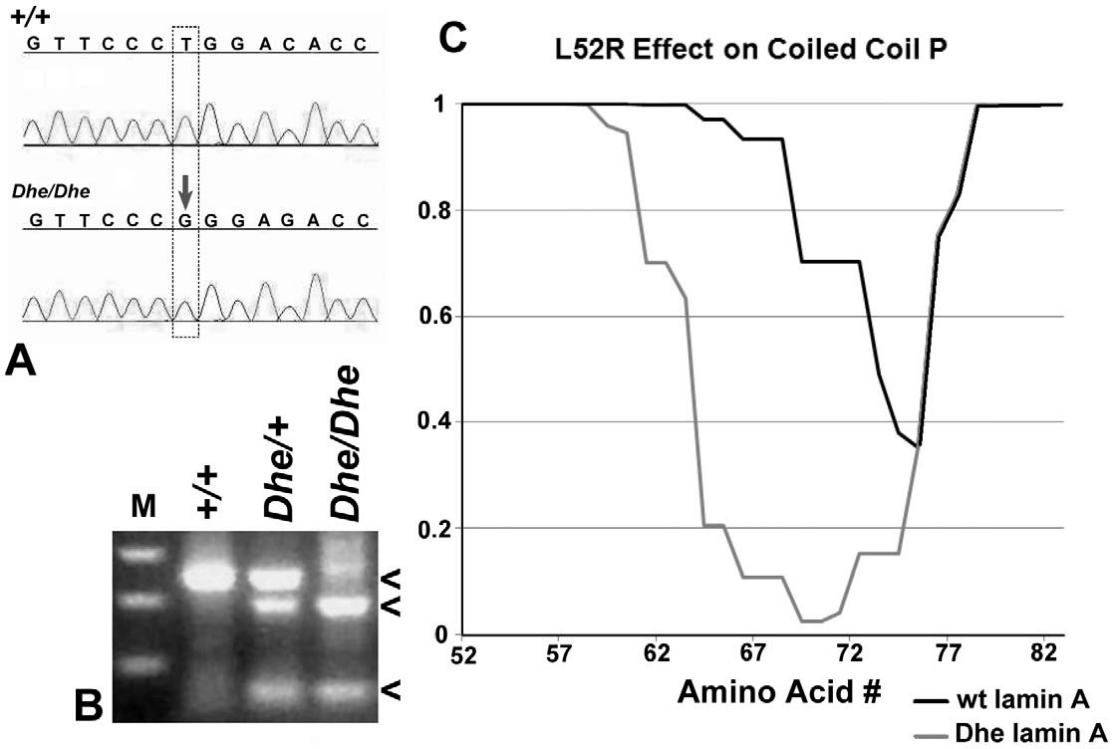
The *Dhe* mutation. (**A**) T to G transversion was identified in exon 1 of *Lmna* in nucleotide 155, starting at the initial A of Met 1, resulting in a change from L to R at amino acid 52 of the protein, early in the coiled coil rod domain. (**B**) A genotyping assay was designed to identify wild type (+/+), heterozygous and homozygous mutants by the gain of a Sma I restriction site in an amplified region of *Lmna*. This produces a single 240 bp band in *+/+* animals (upper arrowhead); the 240 bp plus a 186 bp and a 54 bp band in heterozygotes (lower arrowheads); and only the lower molecular weight bands in *Dhe/Dhe*. (**C**) Plot of effect of L52R on coiled coil probability (*y*-axis) in the region following the mutation (*x*-axis is amino acid number). Wild type lamin A has a short, modest dip in probability, whereas the effect of the L to R mutation destabilizes the coiled coil rod domain much more strongly and for a longer span.

Several candidate genes were chosen within the identified interval. Using standard DNA automated sequencing of the *Lmna* gene, and considering nucleotide A of the ATG translation start codon to be numbered as position 1, we discovered a T155G transversion in exon 1, leading to a missense mutation which substitutes an arginine for a leucine at amino acid 52 (L52R; **Figure 2A**).

The mutation introduced a new SmaI restriction site in the *Dhe* allele, which formed the basis for a genotyping assay. PCR amplification of genomic DNA using primers flanking the point mutation produced a 240 bp product in both wild-type and mutant mice. This product was then digested with the restriction enzyme SmaI, resulting in two fragments of 186 and 54 bp after SmaI digestion, in the presence of the mutant allele. Using this assay, all three genotypes were identified (**Fig. 2B**), consistent with the expected phenotype, confirming this mutation is indeed the *Dhe* mutation. During the crosses performed for mapping, it was noted that the phenotype was consistent in multiple backgrounds.

Analysis of the mouse lamin A with the COILS 2.1 program [13], [14] was performed. The COILS program scans an amino acid sequence for the occurrence of hydrophobic residues along one face of an alpha helix to determine the probability of its forming a coiled coil structure with a similar region of a different protein. The coiled coil rod domain of lamin A begins at amino acid R25. L52 is at position *d* in the heptad repeat pattern, placing it at the eighth helical turn of the rod domain, and on the hydrophobic surface that forms the stable contact between interacting proteins (probability  = 1.000). The substitution of R for L at this position has a dramatic effect on the predicted coiled coil domain in the region immediately following the mutation. **Figure 2C** shows a comparison of coiled coil probability in wild type vs. *Dhe* lamin A in the region immediately following amino acid 52. There is normally a short dip in coiled coil probability beginning at amino acid 66, reaching a low point of 0.35 at residue 75, and returning essentially to P = 1 by residue 79. The *Dhe* mutation is predicted to disturb the coiled coil rod earlier, much more significantly, and over a greater length, causing a drop to P = 0.025 at amino acids 69 and 70. This result strongly suggests that the interactions of *Lmna^Dhe^-* derived proteins could be substantially altered and/or destabilized.

### Abnormal morphology of skull, jaws, and ears and low bone mineral density

Results of digital caliper measurements of craniofacial landmarks in skulls of 12-week-old mice are shown in **Table 1**. A sexual dichotomy was found for both *Dhe/+* and normal mice for most skull measurements, but not for their ratios, indicating that the overall skull morphology was not dependent upon sex. One exception was the ratio of skull length to width, where males of both genotypes (*Dhe/+ and +/+*) appear to have longer, narrower skulls than females. Values were lower for all of the single skull measurements (with the exception of inner canthal distance) for both male and female *Dhe/+* mice compared to controls, suggesting that the overall skull size of *Dhe/+* was reduced, consistent with the smaller body size of *Dhe/+*. The abnormal morphology of the *Dhe/+* skulls was demonstrated by the skull ratios. The jaw length ratio (upper/lower jaw) was greater in *Dhe/+* as would be suggested by their underdeveloped mandible. This inferior brachygnathism was partially offset by a shortened maxilla (superior brachygnathism), but not enough to prevent malocclusion; the skull-to-nose length ratio was greater in *Dhe/+* indicating a shortening of the nose, and both the skull length-to-width and skull height-to-width ratios were less in *Dhe/+*, evidence of a shorter, wider skull. The abnormal jaw length ratio was concordant with malocclusion of the incisors in *Dhe/+* animals, which necessitated weekly trimming the incisors to permit mastication. Cleft palate was not observed.

**Table 1:** Genotype and gender-specific differences in skull morphology of 12-week-old *Dhe*/+ and control skulls stained with alizarin red (mean ± SEM, n = 6 for all groups).

Measurement	*Dhe/*+ Males	+*/*+ Males	*Dhe/*+ Females	+*/*+ Females
Skull Length (mm)	20.87±0.14^B^	22.76±0.36	20.35±0.28^B^	22.77±0.12
Nose Length (mm)	13.51±0.13A, B	14.80±0.29	13.05±0.15^B^	15.27±0.10
Skull Height (mm)	9.30±0.17^B^	10.50±0.17	9.05±0.12^B^	10.32±0.27
Skull Width (mm)	10.41±0.09^B^	11.02±0.03	10.31±0.12^B^	10.82±0.19
Inner Canthal Distance (mm)	5.08±0.11^B^	5.43±0.04	4.88±0.13^B^	5.39±0.10
Lower Jaw Length (mm)	8.51±0.09A, B	11.26±0.08	8.04±0.16^B^	10.91±0.14
Upper Jaw Length (mm)	12.84±0.14^B^	15.37±0.28	12.89±0.21^B^	15.77±0.24
Jaw Length Ratio	1.51±0.02A, B	1.37±0.02^A^	1.61±0.03^B^	1.45±0.03
Skull/Nose Length	1.55±0.01	1.54±0.01	1.56±0.02^B^	1.49±0.01
Skull Height/Length	0.45±0.01	0.46±0.01	0.45±0.01	0.45±0.01
Skull Length/Width	2.01±0.02	2.07±0.03	1.98±0.03^B^	2.11±0.03
Skull Height/Width	0.89±0.01^B^	0.95±0.01	0.88±0.01^B^	0.95±0.01

Note that nearly all of the parameters of craniofacial assessment were significantly different in wild type vs. *Dhe/+* in both males and females.

A p≤0.05 male vs. female within genotype.

B p≤0.05 +/+ vs. *Dhe*/+ within sex.

Ear measurements and DEXA body mass and density measurements are shown in **Table 2**. Pinnae of both male and female *Dhe/*+ mice were significantly shorter than those of +/+ mice. The heterozygous mutants had significantly lower body mass and bone mineral density, both in the total skeleton and in the skull. The areal bone mineral content (g/cm^2^) confirms that the difference in bone mineral is not simply as would be expected for smaller animals, but represents *bona fide* osteopenia. Interestingly, there was a roughly 50% reduction in total body fat in *Dhe/+*, but this difference was restricted to males. *Dhe/Dhe* animals were not examined because accurate DEXA measurements require that animals reach the size attained by wild type animals at about 1 month of age, and the homozygous mutants die at about 10 days.

**Table 2:** Ear pinna lengths (mm) and dual X-ray absorptiometry measurements in 12 week-old *Dhe*/+ and controls (mean ± SEM; n = 6 per group).

Measurement	*Dhe/*+ Males	+*/*+ Males	*Dhe/*+ Females	+/+ Females
Right Ear Pinna Length (mm)	10.26±0.31 B	13.72±0.13 A	10.25±0.27 B	13.26±0.05
Whole Body BMD (g/cm^2^)	0.037±0.0003 B	0.043±0.001	0.034±0.001^B^	0.042±0.001
Total Mass (g)	16.53±0.62 A B	23.63±1.05 A	12.80±0.65 B	17.28±0.84
% Fat	9.417±0.299 A B	18.317±1.619 A	12.183±0.270	13.783±0.796
Skull BMD (g/cm^2^)	0.091±0.001 B	0.104±0.002	0.090±0.002 B	0.105±0.002
Skull BMC (g)	0.170±0.005 B	0.262±0.022	0.158±0.008 B	0.229±0.007
Skull BMD/ Whole Body BMD	2.479±0.013 A	2.425±0.039	2.624±0.047	2.527±0.043

A p≤0.05 male vs. female within genotype.

B p≤0.05 +/+ vs. *Dhe*/+ within sex.

### Patent sutures, defective skull mineralization, and low collagen expression

**Figure 3:**
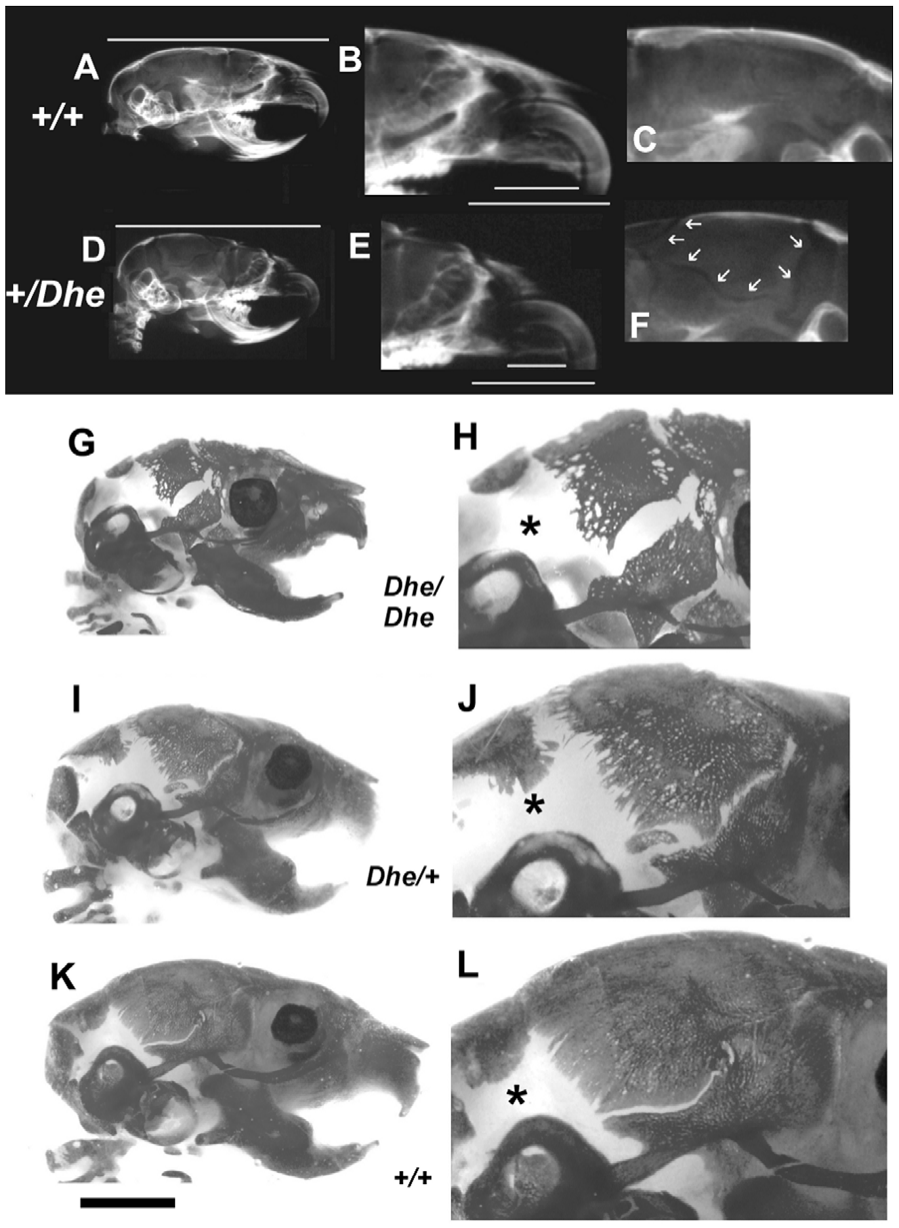
Skull growth and mineralization deficiencies. X-rays of 6 week-old *+/+* (A, B, C) and *Dhe/+* (D, E, F) hemisected skulls. Note lower bone mineral in cranial vault (compare A and D) in the mutant. Total skull length in D is 92% of A (white lines above, A & D). Diastema in E (mandibular arch: the upper white line in B and E) is 67% of that in B, and incisor length in E (the lower white line in B and E) is 87% of that in B. The entire margin of the parietal bone (indicated by white arrows in F) is radiolucent and visible, whereas it cannot be seen C. (G–L), Alizarin red stained skulls of 9-day-old *Dhe/Dhe* (G, H), *Dhe/*+ (I, J), and +/+ (K, L) mice. Overall deficient bone growth and mineralization are evident in *Dhe/Dhe* and *Dhe/+* skulls. Right-hand panels are slightly enlarged views of the skulls in the left panels. Note deficient skull length and failure of mineralization to proceed, especially in the cranial vault, with large, open areas around the parietal and occipital bones (*).Bar in K = 9.6 mm in A, D; 4.1 mm in B, E; 0.76 mm in C, F; 2 mm in G, I, K; and 0.94 mm in H, J, L.

**Figure 4:**
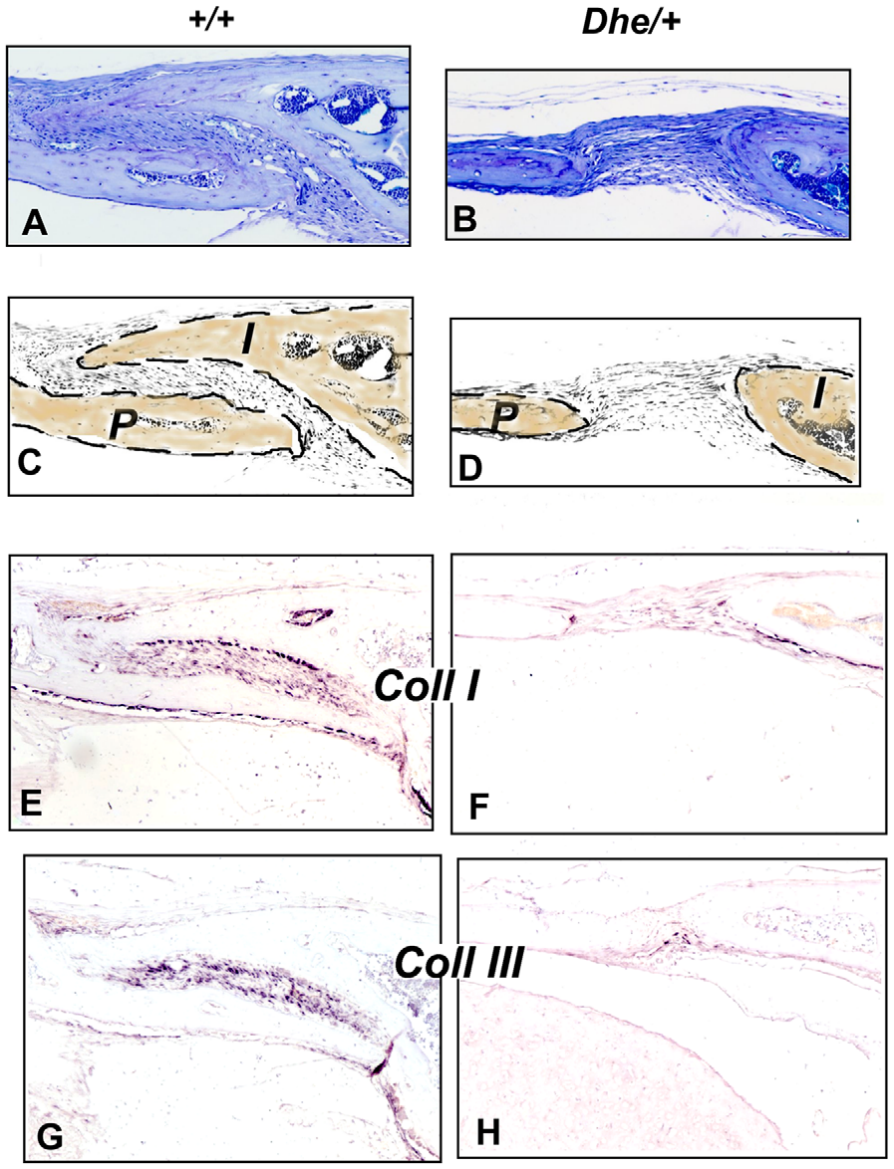
Cranial suture histology and collagen expression in +*/*+ and *Dhe/*+ mice, 6 weeks post partum. **A, C, E, G**, *+/+* genotype; **B, D, F, H**, *Dhe/+* genotype. Lateral views of the coronal suture, 5 µm paraffin sections. A, B, toluidine blue stain. C, D, diagrams of A and B with the parietal (*P*) and interparietal (*I*) bones outlined and highlighted. E–H, i*n situ* hybridization of adjacent sections for collagens type I (E, F) and type III (G, H). Original magnification  = 100X. Intensity of the purlish color indicates relative level of the mRNA.

Radiographs of 6-week-old +/+ and *Dhe/+* skulls are shown in **Figure 3**. The reduced bone mineral density is evident in the overall greater radiolucence of the cranial vault and the anterior portions of the skull (compare **3A** and **3D**). The maxillary incisor and the diastema (the maxillary arch) are both abnormally short in the mutant (compare **3B** and **3E**). Note also that the entire outline of the parietal bone is visible in the mutant, indicating patent sutures (compare **3C** and **3F**). Also shown in **Figure 3** are Alizarin red stained, clarified skulls of 10-day-old pups of all 3 genotypes. There is a striking deficiency in both hetero- and homozygous mutant skulls in bone growth and mineralization. Large, open areas dominate the posterior aspect of the skulls, with *Dhe/Dhe* having a much more pronounced growth and mineralization defect (**3G–L** ****). The skull defects persist in *Dhe/+* mice, as seen by histological examination (**Figure 4A, B**). The suture joining the parietal and interparietal bones (i.e., the coronal suture) is closed in the 6-week-old *+/+* mouse (**4A**), with overlapping bone plates joined by dense connective tissue. In the *Dhe/+* mouse (**4B**), the bones do not overlap, and the suture tissue is hypoplastic, forming a much looser, thinner connection of the plates (in **4C** and **4D**, the bone outlines and identification are diagrammed). These same abnormalities were present in all cranial vault and facial sutures examined, including the frontal, lambdoid, and pre-maxillary/maxillary sutures (not shown).

**Figure 5:**
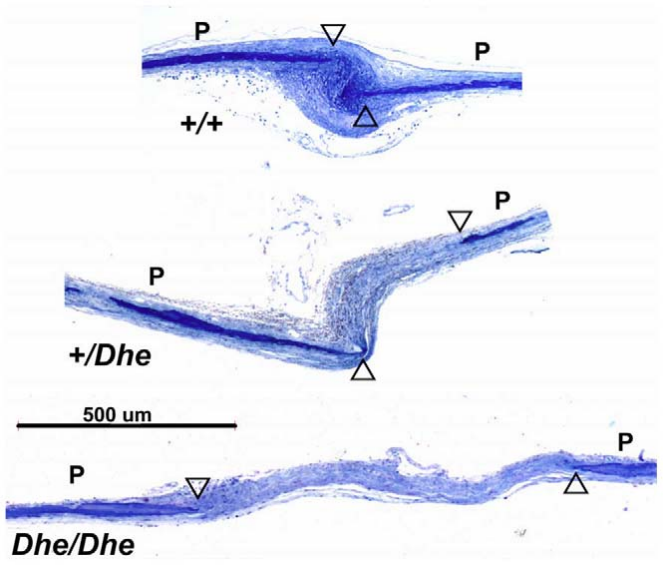
Sagittal sutures in 1-week-old mice. +/+ mouse skull shows normal suture development (top), whereas heterozygous (middle) and homozygous (bottom) *Dhe* mice have deficient cranial growth. The +/+ skull shows normal histology, with the edges of the parietal bones (P) nearly overlapping (open arrowheads) and a dense growth of connective tissue surrounding the edges of the bones. In *Dhe/+*, the bones do not reach one another, the bones themselves are thin, and the connective tissue between them is less dense. In *Dhe/Dhe*, the phenotype is more severe, with a suture nearly a millimeter wide and the connective tissue in the gap loose and disorganized. 2 µm Epon sections stained with toluidine blue.

*In situ hybridization* (ISH) was performed to determine whether major mRNA species for extracellular matrix would reflect the tissue abnormalities (**Figure 4E–H**). The mRNA for type I collagen, the major protein component of bone, was robustly expressed in osteoblasts on the bone surface in the *+/+* mice (**4 E**), whereas it was barely detectable in *Dhe/+* mice (**4 F**). Similarly, the centro-sutural cells in *+/+* mice had very high levels of type III collagen mRNA (**4 G**), consistent with synthesis of a tough, fibrous suture. Again, in *Dhe/+* mice, type III collagen mRNA was nearly absent, indicative of deficient connective tissue formation. (**4H**). Histological assessments of skulls of all three genotypes, *+/+*, *+/ Dhe*, and *Dhe/Dhe*, were done at one week post partum. Frontal sections of the skulls (**Figure 5**) revealed normal sagittal suture morphology in +/+ mice (**Fig. 5, top**), with bones slightly misaligned to permit overlap during birth, and a robust growth of connective tissue between and around the bones' ends. In contrast, the *Dhe/+* mice (**Fig. 5 middle**) had a gap of approximately 500 µm between the edges of the parietal bones and these bones were connected by a much thinner band of connective tissue. In the *Dhe/Dhe* mice (**Fig. 5 bottom**), the gap between the parietal bones was approximately a millimeter, and the band of connective tissue in the suture was thinnest of all the three genotypes.

### Changes in hair, skin and oral epithelia

**Figure 6:**
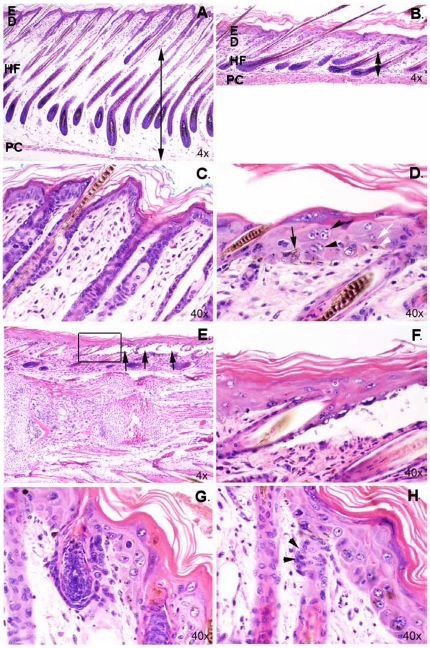
General histology demonstrating skin lesions of *Dhe/Dhe* mouse. (**A** and **C**) Wild-type trunkal skin sections illustrating well organized epidermis (E) and dermis (D), as well as anagen stage hair follicles extending through hypodermal fat (HF) region to the panniculus carnosus muscle (PC). (**B** and **D**) *Dhe*/*Dhe* trunkal skin sections exhibit a reduced hypodermal fat layer (double arrow in panels **A** and **B**), thickened epidermis, orthokeratotic hyperkeratosis, multinucleated keratinocytes in stratum spinosum (black arrow heads in panel **D**) and interfollicular epidermal pigmentation in dendritic cells (black arrow in **D**) and keratinocytes (white arrow in **D**). Apoptotic keratinocytes are scattered throughout stratum basale (white arrowhead in **D**). (**E**) *Dhe/Dhe* tail skin sections with large areas of dermal-epidermal separation (arrows). (**F**) Higher magnification image of boxed portion of panel **E** showing beginning of epidermal-dermal separation. (**G** and **H**) *Dhe/Dhe* trunkal skin section with shortened anagen phase hair follicles limited to dermis and hypoplastic sebaceous glands (arrow heads in panel H). (6 µm sections of 9-day-old mice; original magnifications shown in each panel).

**Figure 7:**
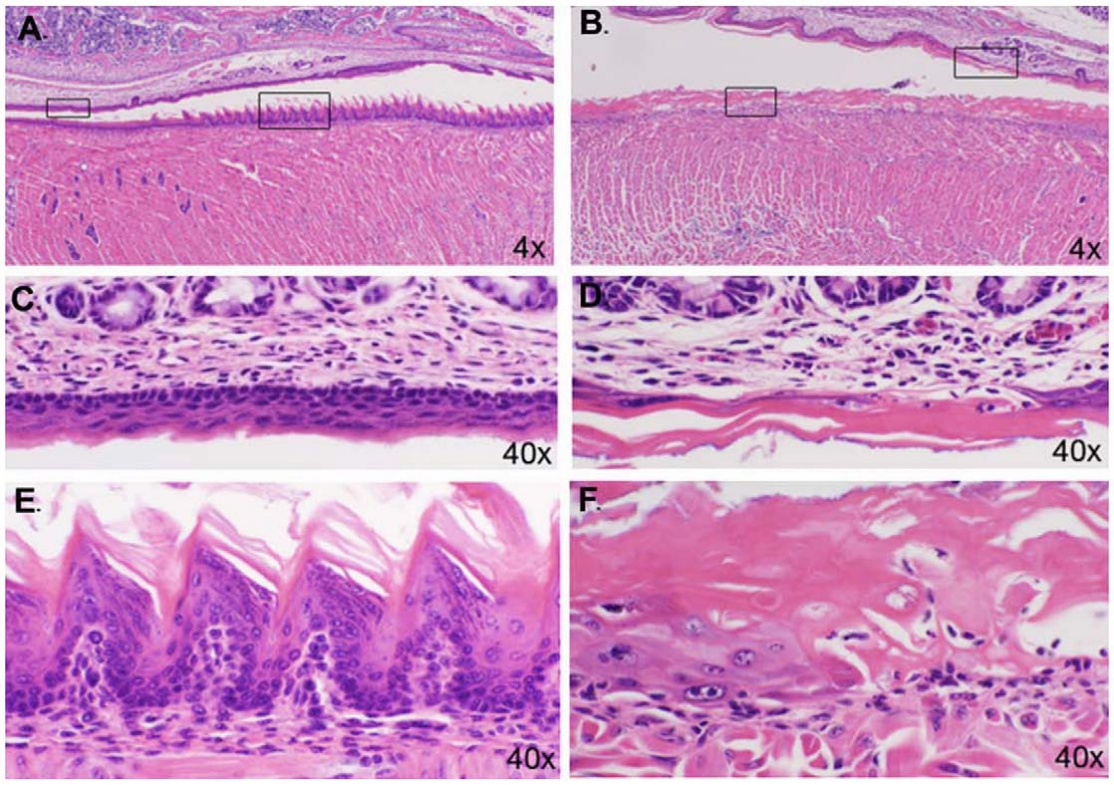
Oral ulcers in *Dhe/Dhe* mice. (**A, C** and **E**) Sagittal sections of +/+ tongue (**A** and **E**) and hard palate (**A** and **C**) demonstrate the normal terminal differentiation of these stratified squamous epithelia. (**B, D** and **F**). Comparable sections from a 10 day old male *Dhe/Dhe* tongue (**B** and **F**) and hard palate (**B** and **D**) revealed the dysplastic epithelium (**F**) at the edge of an extensive ulcer (**B**). Boxes in **A** and **B** outline areas shown at higher magnification in **C** and **E**, and **D** and **F**, respectively.

In both heterozygous and homozygous mutant mice, hair color was light compared to the black controls, and *Dhe/Dhe* mice had diffuse alopecia. Photomicrographs of skin are shown in **Figure 6 A–H**. At 9 days of age, +/+ mice had normal dorsal thoracic skin (**Fig. 6A, C**). Hair follicles were in the late anagen stage of the embryonic hair cycle. The hypodermal fat layer was thick and hair bulbs extended deep into the fat layer to just above the panniculus carnosa muscle (**Fig. 6A**). The epidermis was slightly thicker than that found in adults, which is normal for this age. By contrast, the *Dhe/Dhe* skin had hair follicles in the same stage of the hair cycle but the hypodermal fat layer was markedly thinned (**6B, D**). The dermis was of comparable size to that of the +/+ mice; however, the epidermis was abnormally thickened. This was due both to more cells (hyperplasia) and also to increased cell size (hypertrophy). Many keratinocytes were binucleated (**6 D**). Cells in the stratum granulosum were prominent with abundant cytoplasm with a fine basophilic stipling. This was in sharp contrast to the +/+ mice in which these cells were difficult to identify and often were seen only as very thin, flattened, dark blue streaks below the stratum corneum. Normal mouse skin lacks interfollicular epidermal pigmentation. *Dhe/Dhe* mice had dentritic cells containing pigment within the interfollicular epidermis (**6D**). These epidermal changes are suggestive of a preneoplastic condition, epidermal dysplasia. While many hair follicles appeared to be normal, there were scattered follicles that were unusually short with the bulb located immediately below the epidermis (**6G, H**). These did not produce normal hair shafts. The skin covering the tails of these mutant mice (**6E, F**) was similar to the dorsal skin. Mouse tail skin is normally much thicker than trunkal skin. However, the tail skin epidermis separated along the level of the basement membrane resulting in ulcer formation. Similar changes were evident in the oral mucosa of the tongue and hard palate (**Figure 7**). These are highly specialized structures. In the *Dhe/Dhe* mice, there was locally extensive coagulative necrosis with severe ulcer formation on the tongue (**7B, F**) and separation of epidermis from dermis on the hard palate (**7D**). These changes would interfere with the mouse's ability to eat and probably contributed to the severe runting. In addition, extensive ulceration of the tail skin and oral mucosae would result in fluid and electrolyte loss leading to death. Changes in the skin were minimal in the *Dhe*/+ mice, primarily scattered follicular dystrophy similar to that seen in B6 strain specific alopecia and dermatitis, making it difficult to separate a specific phenotype in these mice with strain specific background lesions.

### Abnormal morphology of osteoblast nuclear lamina

**Figure 8:**
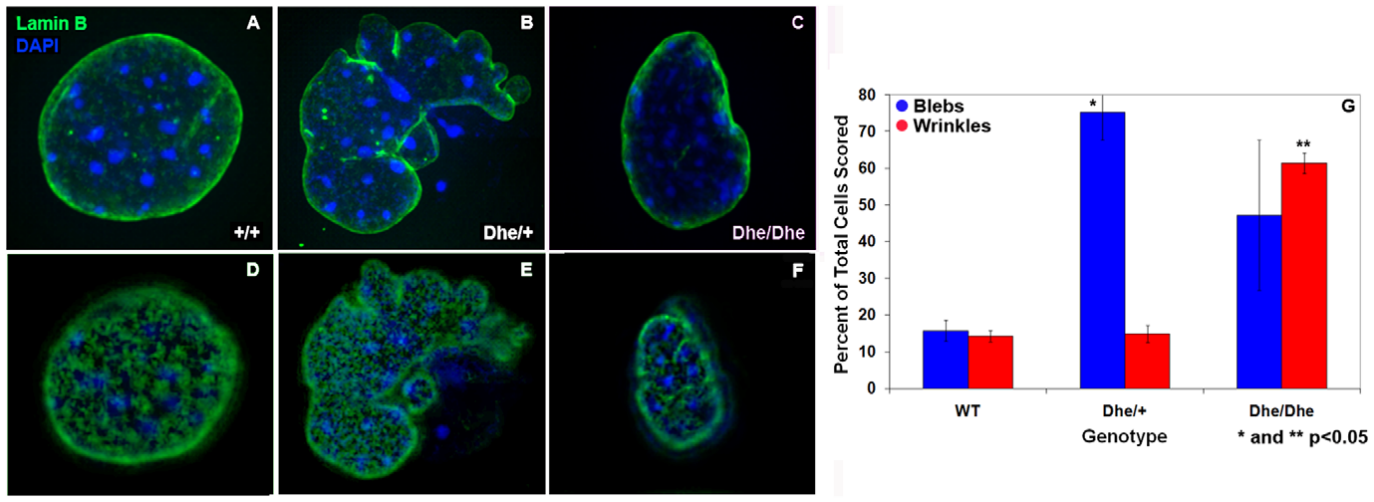
Genotype specific nuclear envelope deformations in *Dhe* mouse clavarial osteoblasts. (**A–F**). Immunolabeling with anti-lamin B (green) and DAPI counterstain (blue) reveals the extent of nuclear lamina deformation in images of a single optical section through the mid-nucleus of cultured wild-type (+/+) (**A**), heterozygous (*Dhe/*+) (**B**) and homozygous (*Dhe/Dhe*) (**C**) primary osteoblasts. (**D–F**) Top-most single optical sections of representative cells for each *Dhe* genotype demonstrating the genotype specific dysmorphologies of the nuclear lamina. (**G**) Anti-lamin B labeled +/+, *Dhe*/+ and *Dhe/Dhe* cells were imaged in three-dimensions and scored for the presence or absence of one or more blebs (blue bars) and/or wrinkles (red bars) in the nuclear lamina. Scoring indicated that blebbing occurs more frequently in *Dhe*/+ osteoblasts, while wrinkled nuclear envelopes are observed preferentially in *Dhe/Dhe* cells as compared to wild-type mice (* and ** students t-test p-value <0.05).

The nuclear morphology of cultured primary calvarial osteoblasts from *Dhe* mice was examined by indirect immunofluorescence to determine the extent of deformation of the nuclear lamina. As seen in **Figure 8**, optical sections through the middle (**Fig. 8A**) and top (**Fig. 8D**) of wild-type mouse osteoblasts reveal a continuous, smooth nuclear lamina as detected by lamin B1 immunolabeling. In contrast, the heterozygous and homozygous *Dhe* cells demonstrate grossly abnormal nuclear lamina. The heterozygous mutants exhibit various numbers of nuclear lamina blebs, discreet balloon-like outpocketings of the nuclear envelope (**Fig. 8, B** **** and **E**, middle section and top section, respectively). Quantification of nuclear blebbing indicated a statistically significant increase in blebs in heterozygous mutants compared to wild-type or homozygous mutants (p<0.05, Student's *t*-test, **Fig. 8G**, blue bars). Homozygous *Dhe* cells exhibit a dysmorphic nuclear lamina as well, but the phenotype is quite different from the heterozygote phenotype. Homozygous mutants show distinctive wrinkling or furrowing of the nuclear lamina as compared to wild-type controls (**Fig. 8**, **C** and **F**). Although wrinkling was observed in a limited number of cell in the other genotypes (panel **G**, red bars), furrowing was greatly increased in homozygous cell nuclei (p<0.05, Student's *t*-test, **Fig. 8 G**, red bars). Therefore, *Dhe* osteoblast nuclei exhibit genotype-specific manifestations of a novel, spontaneous mutation in the *Lmna* gene.

## Discussion

The *Dhe* mutation is the first spontaneous mutation in the *Lmna* gene in the mouse. Our investigations to date have centered on abnormalities in skull, bone, skin, and fat and on the nuclei of cultured connective tissue cells. It seems likely that other tissue and organ systems are affected, but to date we have not investigated them sufficiently to be certain. The findings reported here show that *Dhe* allele imparts a variety of phenotypes that are also seen in human laminopathies. These include craniofacial abnormalities resembling those in mandibuloacral dysplaisa (MAD) and Hutchinson-Gilford progeria syndrome (HPGS), defects of the skin and hair found in restrictive dermopathy and HPGS, and markedly reduced hypodermal fat, reminiscent of human lipodystrophies. Similar to many human laminopathy alleles, the *Dhe* mutation also causes defects in heterozygous mice. However, unlike in human lipodystrophy, no primary adipocyte abnormality was found in *Dhe* mutants, and it is likely that poor nutrition as a consequence of abnormal jaw morphology contributes to the lack of body fat in *Dhe* heterozygous mutants. Nutritional deficits are extremely likely in *Dhe/Dhe* animals due to the considerable defects in the oral lining (**Figure 7**), and we suspect that this, combined with fluid loss through ulcerations in the skin and oral lining may be causative of death in homozygous mutants. Interestingly, nuclear architecture differed between heterozygous and homozygous *Dhe* mutant cells. Given the dose-dependence and range of its phenotypic manifestations, the *Dhe* mouse should provide a useful model for investigations of the etiology of human laminopathy syndromes, and perhaps for gene-therapeutic interventions.

*Pathogenesis of laminopathies remains unresolved*. No clear-cut mechanism has been determined for the pathogenesis of any laminopathy in any species; however, it is predicted that disrupted lamin A/C structure and protein/protein interactions underlie the various laminopathic syndromes. Unlike other intermediate filament family proteins, the lamins lack a flexible linker domain in the amino terminal half of the α-helical rod. The linker regions are thought to provide flexibility and to permit unwinding of the N-terminal 35 amino acids of the rod, designated segment 1A [15], presumably to facilitate its reorganization, self-assembly, and interaction with other proteins. The COILS prediction program scans an amino acid sequence to determine the probability of coiled coil formation by identifying heptad repeat patterns of hydrophobic amino acids at the first and fourth position of an α-helical region, combined with basic and acidic residues at adjacent positions, which together to promote ionic stabilization of the hydrophobic inter-protein interaction [13], [14]. The position of the mutation in *Lmna^Dhe^* at amino acid 52 places it in a critical spot for hydrophobicity-based coiled coil protein-protein interaction early in the rod domain. The disruption of the normal coiled coil rod domain (**Figure 2** **C**) in *Dhe* is a likely contributor to the phenotype. The destabilization of the heptad repeat in lamin A/C by the L52R mutation may in effect introduce a *de novo* flexible “linker” into the affected proteins to disrupt their normal self- and/or non-self associations. Interestingly, it was recently been shown that not all mutations associated with human disease affect filament assembly [16]. *Caenorrhabditis elegans* carries only a single lamin gene, and point mutations were introduced which align with residues that cause human laminopathies. Only 4 of 14 such mutations affected assembly of *C elegans* lamin into filaments or paracrystals.

A growing list of proteins (and DNA) have been found to interact with different domains of lamin A/C. In a recent review, it was proposed that the binding partners may be grouped into four broad categories: architectural (e.g., lamin B, actin); chromatin (e.g., histones, DNA minor groove); gene regulation (e.g. pRb, SREBP1 a/c); or signaling (e.g., 12-S lipoxygenase, PKCα); and others with unknown roles (e.g., nuclear prelamin A recognition factor, or NARF) [1]. These groupings alone are enough to indicate a broad range of potential functions for lamin A/C in genome organization and function. The role of the NL is also being seen to extend beyond the nuclear periphery, especially via its interactions with nuclear pores and transmembrane proteins that connect the nuclear interior with the cytoskeleton, implying extra-nuclear structural roles that may affect cell function and viability [17].

There is as yet no simple story to account for which tissues may be impacted by a particular mutation. Jacob and Garg recently reviewed the plethora of human mutations, spread throughout the *LMNA* gene, which can cause similar symptoms [6]. For example, point mutations causing single amino acid substitutions in 5 different exons, 1, 7, 8, 9, and 11, are associated with lipodystrophies. Even more remarkably, single amino acid substitutions in exons 1–11 are causative of EDMD. Dilated cardiomyopathy (DCM) is caused by point mutations which have been mapped to exons 1, 2, 3, 4, 6, 8, 9, 10, and 11. HGPS has been mapped to mutations in exons 7 and 11. Each of the mutations may or may not also be associated with co-existing tissue defects [6]. In progeroid mutations, genomic instability, checkpoint delay, and aneuploidy were postulated to result from an inability to recruit DNA repair factors 53BP1 and RAD51 of the p53 pathway [18].

### Mouse models of laminopathies and correlation to human disease

Lamin A–C knockout mice are born appearing normal, but are growth retarded and develop cardiac and skeletal muscular dystrophies and demyelination of the sacral nerve, and die at 6–7 weeks post-partum [19]. Intriguingly, a lamin C-only gene replacement mouse, which yields mice with no lamin A but only lamin C, has none of the dystrophic syndromes associated with either the complete knockout of *Lmna* (which necessarily also deletes lamin C) or of mutations in portions of the gene that are shared by both A and C [20]. It may be inferred that either the A or C product of *Lmna* is required for survival, but not both.

Mouse models intended to mimic human laminopathies have not always yielded precisely the anticipated phenotype. For example, mice with a L530P mutation, which causes AD-EDMD in humans, were found to more closely model HGPS [21]. The elimination of exons 8–11 causes the loss of lamins A and C, but is not embryonic lethal, leading rather to muscular dystrophy, DCM, and death by the 8^th^ postnatal week [19]. Another mouse model with a specific missense mutation introduced into *Lmna* (H222P), which causes AD-EDMD in humans, was found to have adult-onset skeletal and cardiac muscular dystrophy, with penetrance more severe in males than females [22]. Thus, attempts to produce faithful models of human laminopathies in mice have sometimes produced unanticipated results, reflecting the complexities of these disorders and their underlying mechanisms.

Although enlarged fontanelles have been described in restrictive dermopathies in human patients [23], we are not aware of laminopathies in which cranial dysostosis has been reported to be a major symptom; however several syndromes do impact the skeleton. Osteopenia, acral bone loss and/or osteoporosis are associated with MAD or HGPS. The shortened jaws, osteopenia, and dermal hypoplasia in *Dhe* are reminiscent of MAD in humans [9], and the lack of body fat in *Dhe* is consistent with that seen in familial partial lipodystrophy [24], [25]. Whether there is subtle loss of distal bone in extremities in *Dhe* remains to be determined, but X-ray analyses and alizarin red staining of whole skeletons suggest no visible foreshortening of the bone. It will be important to determine whether the lack of growth in the cranial vault in *Dhe* is a primary or secondary effect. For example, the importance of the dura in providing growth signals to the calvaria is well known [26], [27]. The cranial dysplasia in *Dhe* could be the result of loss of growth signals from the dura, or it may be that the cells of the calvaria are unable to respond to dura-derived signals.

The pathogenesis of laminopathic syndromes remains elusive, and many basic questions are unanswered. For example, what role might modifier genes play in aspects of the phenotypes seen? For the *Dhe* mutation, the phenotype was consistent in multiple backgrounds, including the crosses that were done to map the mutation. Crossing mice having laminopathic mutations with other strains carrying alleles of lamin-associated proteins may be one route to understanding some underlying mechanisms. Is the hypoplasia of specific tissues due to cell death, failure of proliferation, lack of differentiation, or some combination of these? The distortions of nuclear morphology in *Dhe* cells indicate an effect at the sub-cellular level that varies with gene dose (**Figure 8**). Interestingly, heterozygous nuclei exhibited blebbing, and few wrinkled nuclei were seen, whereas *Dhe/Dhe* nuclei had both blebbing and wrinkling of the nuclear surface. Thus, gene dose contributed to the type and extent of nuclear distortion present.

While it has been proposed that laminopathies reflect cellular damage due to fragility in tissues that are subject to mechanical stress, the results shown here also suggest inherent cellular defects. Skin has been shown to be affected in certain laminopathies, from relatively mild effects such as loss of fat and dermal support that may cause thin, papery-looking skin to restrictive dermopathies, which are lethal, and have fragile, easily-torn skin [6], [7], [23]. *Dhe/Dhe* mice exhibit multinucleated and apoptotic epidermal cells in the skin and ulcerations and other defects in the gingiva and lingual mucosae, in addition to their other abnormalities. The impact of the *Dhe* mutation at the organismal level is much more severe in homozygotes, as can be seen in many tissues and in the perinatal lethality of homozygosity vs. the normal lifespan of the heterozygotes. We anticipate that the *Dhe* mouse will provide a useful model in investigations into the etiology and underlying mechanisms of laminopathies.

## Materials and Methods

### Mice

The spontaneous mutation (*Dhe*) was discovered in 1989 at The Jackson Laboratory (JAX, Bar Harbor, Maine), in the BXD8/TyJ recombinant inbred line of mice maintained in the laboratory of Dr. Benjamin Taylor. Upon his retirement, Dr. Taylor donated the mutant strain to the Craniofacial Resource at JAX. Mutant *Dhe* mice were identified by their sparse hair coat and short ears. Subsequently, mating of an affected BXD8/TyJ male to a C57BL/6J wild-type female, followed by intercrossing of affected F1 mice revealed a semi-dominant autosomal mode of inheritance wherein homozygous *Dhe/Dhe* mice are viable for approximately ten postnatal days. Mapping data revealed that the *Dhe* mutation arose within a C57BL/6J segment of the BXD8/TyJ strain, and the mutation has now been made congenic on the C57BL/6J inbred strain through repeated backcrosses (N50F1) and, subsequent to causative gene identification, has been named B6.BXD8-*Lmna^Dhe^*/TyJ.

Mice for this work were produced by The Craniofacial Resource at JAX and were maintained under 14∶10 hour light:dark cycles; autoclaved diet NIH-31 (6 percent fat, 18 percent protein, Ca:P 1∶1, vitamin and mineral fortified; PMI, Richmond, IN) and HCl acidified water (pH 2.8–3.2) were provided *ad libitum*. Mice were housed in groups of 4 or 5 within polycarbonate boxes of 51 square inch area on sterilized shavings of Northern White Pine as bedding. Serum was harvested and skeletal preparations were made at necropsy. All procedures were approved by The Jackson Laboratory's Institutional Animal Care and Use Committee and performed in accordance with National Institutes of Health guidelines for the care and use of animals in research.

### Genetic mapping, gene identification, and genotyping assay development

Following initial mapping to Chromosome 3 by the Taylor lab, the *Dhe* mutation was fine-mapped in the Craniofacial Resource at The Jackson Laboratory. Heterozygous *Dhe/+* mice were mated to mice of the wild-derived strain CAST/EiJ, chosen for genetic polymorphism with C57BL/6J mice, the strain of origin of the *Dhe* mutation. Affected F1 hybrids were then crossed to C57BL/6J, and 256 affected backcross (BC1) mice were produced. DNA was isolated from frozen spleens of all affected BC1 mice using phenol/chloroform extraction and typed for polymorphic markers throughout the genome using standard PCR methodology [28]. Gene order and recombination frequencies were calculated with the Map Manager computer program [29].

Candidate genes were selected within the chromosomal region identified for the *Dhe* mutation. Primers flanking each exon of each of the candidate genes were used to amplify genomic DNA derived from homozygous *Dhe/Dhe* mice by standard PCR, and then subjected to DNA sequencing using the same primers. A 3700 DNA sequencer (Applied Biosystems, Foster City, CA) was used with an optimized Big Dye Terminator Cycle Sequencing method.

Once the causative gene was identified, a genotyping assay was designed to detect the *Dhe* mutation. PCR primers flanking the *Dhe* mutation located in exon 1 of the mouse *Lmna* gene were used to amplify a 240 bp product from genomic DNA. These primers are: dhefwd 5′- ACCTGCAGGAGCTCAATGAC-3′, dherev 5′ –TGAACTCCTCACGCACTTTG – 3′. PCR products were then digested with SmaI for 4 hours or overnight at 37 C and resolved on 1.5% NuSieve agarose gel (Cambrex Bio Science, Rockland, ME).

*Coiled coil prediction:* The COILS 2.1 prediction program [13], [14] was used with a 28 amino acid window without weighting to analyze the amino acid sequence of mouse wild type and *Dhe* lamin A (GenBank accession #BAA08569).

### Skull morphometry

Skulls were skinned, cleaned of extraneous tissue and placed in 1% KOH plus a few drops of 5% Alizarin red S (Sigma) for 24–48 hours, after which they were placed in 1% KOH until maceration was complete. Skulls were then transferred to a 2∶2∶1 mixture of 70% ethanol, glycerol, benzyl alcohol for up to 24 hours to clarify, and stored in 1∶1 70% ethanol/glycerol. Digital calipers (Stoelting, Wood Dale, IL) were used to measure a series of key craniofacial dimensions of alizarin red-stained skulls from *+/+* and *Dhe/+* mice at 12 weeks of age. Landmarks for skull measurements were adapted from those used by Richtsmeier to characterize craniofacial morphology in mouse models of Down Syndrome [30] and have been validated for accuracy and precision in our hands [31]. Measurements were made of 6 animals of each sex and genotype.

### Radiographic and skeletal histological studies, including in situ hybridization

Skulls were hemisected and radiographed (Faxitron Micro 50, Wheeling, IL) using the same exposure settings for all animals of a given age [32]. Radiographs were digitized using a flatbed scanner (Microtek 9800 XL, Carson, CA)) using identical brightness and contrast settings for all radiographs. For histological studies and *in situ* hybridization, hemisected skulls were fixed overnight in 4% paraformaldehyde, demineralized in EDTA, paraffin embedded, and processed for *in situ* hybridization (ISH) as described previously [33], [34]. Riboprobes for ISH for collagens type I (α1(I)) and III (α1(III)) were prepared using rat cDNA clones as templates for *in vitro* transcription, incorporating digoxigenin-UTP (Roche, Indianapolis, IN) [33] by *in vitro* transcription using the Ampliscribe T7 kit (Epicentre, Madison, WI) [35]. 5 µm sections were cut and used either for ISH, or were stained with 0.1% toluidine blue for histological visualization. All genotypes were processed together to eliminate variation due to timing, reagent concentration, temperature, etc. For plastic embedment, calvariae from 1-week-old mice were dissected, cleaned of extraneous tissue, fixed with 2.5% glutaraldehyde, demineralized with EDTA, and embedded in Epon (Polysciences, Worthington, PA) as previously described [32], [36]. 2 µm sections were cut and stained with toluidine blue. Microscopic images were obtained with a Zeiss Axioskop 2 equipped with a Zeiss Axiocam HR color digital camera controlled with Axiovision Software (version 4.0).

### DEXA analyses of bone mineral and body fat

Data were collected using the PIXImus small animal DEXA (dual X-ray absorptiometry) system (GE LUNAR, Madison, WI), software version 1.43.036.008. The PIXImus was reconfigured with lower X-ray energy than in human DEXA machines in order to achieve contrast in small specimens. This device measures bone mineral density (BMD) per unit area (g/cm^2^; areal BMD) as well as the mass of body fat per total body mass (% body fat). The resolution of the PIXImus is 0.18×0.18 mm pixels with a usable scanning area of 80×65 mm, allowing for measurement of single whole mice and collections of isolated specimens. The PIXImus was calibrated with a phantom utilizing known values, and a QA is performed daily with this same phantom. Assessment of accuracy for the PIXImus was done with a set of hydroxyapatite standards (0–2,000 mg), yielding a correlation of 0.999 between standards and PIXImus measurement of mineral. The precision for BMD is less than 1% for whole body, approximately 1.5% for specialized regions. Data obtained in this manner correlate strongly with peripheral quantitative computerized tomography (pQCT) values. For example, for 614 isolated spinal vertebrae, correlation was highly significant (p<0.001; r = .704). These data (not shown) were acquired from euthanized mice.

### Cell Culture and Immunofluorescence

Primary cranial osteoblast cultures were obtained using neonatal wild-type, heterozygous and homozygous *Dhe* mice as previously described [37]. Briefly, calvariae from neonatal mice were isolated from dissected crania and subjected to sequential digestions of 5, 15, and 25 min at 37°C in a shaking incubator with 2% Collagenase-P (Roche, Nutley, NJ)/0.25% Trypsin (Mediatec, Herndon, VA). The harvested cells from each individual pup were plated on two 22 mm×22 mm Gold Seal Cover Slips in Earle's Minimal Essential Medium (EMEM; Mediatec) supplemented with 10% fetal bovine serum. Cells were incubated at 37°C with 5% CO_2_ changing media every 2 days for 6 to 9 days. Subsequently, cells were fixed for 10 minutes with 4% formaldehyde and permeabilized for 10 minutes with 0.5% Triton X-100 in 1X Phosphate Buffered Saline (PBS). Next, cells were labeled with goat anti-lamin B1 antibody (clone M20, Santa Cruz Biotechnology, Santa Cruz, CA) followed after washing with Alexa Fluor 488–goat anti–mouse IgG (Invitrogen, Carlsbad, CA). Cells were counterstained with 1 µg/ml 4,6-diamidino-2-phenylindole (DAPI) and mounted in phenylenediamine glycerol.

### Nuclear morphometry

Three-dimensional (3D) images of osteoblast nuclei were acquired using a Zeiss Axiovert 200 M inverted microscope equipped with a Plan-Apochromat 100x/1.40 Oil objective (Carl Zeiss Microimaging, Inc., Thornwood, NJ) along with a Hamamatsu ORCA-ER digital camera (Hamamatsu Photonics, Bridgewater, NJ). Optical sections were acquired at 200 nm intervals. 3D wide-field epi-fluorescence images were deconvolved using AutoDeblur software (Media Cybernetics, Bethesda, MD). Anti-lamin B immunolabeled cell nuclei were scored for the presence or absence of one or more blebs and/or wrinkles in the nuclear lamina using cells from three mice from different litters for each genotype. “Blebs” were defined as in Cappell and Collins [38] to be protrusions from the surface of the nucleus, usually roughly hemispherical in appearance. Wrinkles were defined as sharp concavities in the normally smooth convexity of the nuclear envelope. All optical sections were examined for blebbing and wrinkling by two independent investigators.

### Statistical tests

Data are presented as means ± SEM. Statistical analyses were performed with StatView 4.5 (Abacus Concepts, Berkeley, CA) software for Macintosh. Results for each measured parameter were analyzed by three-way analysis of variance (ANOVA) with gender, time and genotype as the variables. Results were considered significantly different at p<0.05.

### Histopathologic analysis

Mice were euthanized by CO_2_ asphyxiation using IACUC approved procedures. Complete detailed necropsies were performed [39]. Tissues were fixed in Fekete's acid alcohol formalin overnight then stored in 70% ethanol until trimmed, processed routinely, embedded in paraffin, sectioned at 6 um, and stained with hematoxylin and eosin. Slides were reviewed by experienced mouse histopathologists (TI and JPS).
